# Work-family conflicts and long-term medically certified sickness absence due to mental disorders – a follow-up study of female municipal employees

**DOI:** 10.1186/s12889-023-16075-y

**Published:** 2023-06-13

**Authors:** Leena Kaila-Kangas, Eija Haukka, Tea Lallukka, Ossi Rahkonen, Salla Toppinen-Tanner, Päivi Leino-Arjas

**Affiliations:** 1 Specialized Researcher, Finnish Institute of Occupational Health, 00032 Työterveyslaitos, Box 18, Helsinki, Finland; 2 Department of Public Health, University of Helsinki, Helsinki, Finland

**Keywords:** Family, Mental health, Role conflict, Sick leave, Wellbeing, Work

## Abstract

**Background:**

. Decreased work ability due to mental disorders is a growing concern in Europe. We studied the role of work-family conflicts in association with long-term sickness absence due to mental disorders (LTSA-MD).

**Methods:**

. Baseline data were extracted from the Helsinki Health Study for women aged 40 to 55 in full-time work in 2001 − 2002 (N = 2386). Questionnaire responses were linked with register data from the Social Insurance Institution of Finland on SA spells due to mental disorders during 2004–2010. We studied an overall question on satisfaction with combining work and family (WFS) and composite scores of work-to-family conflicts (WTFC) and family-to-work-conflicts (FTWC), and their components in association with the first certified SA spell (≥ 12 calendar days) due to a mental disorder during the follow-up. We performed Cox regression analyses with hazard ratios (HR) and their 95% confidence intervals (CI) adjusted for sociodemographic factors, work schedule, perceived mental and physical strenuousness at work, and self-rated health. First, we examined all participants, and second, only those who reported no prior mental disorder.

**Results:**

. Poor work-family satisfaction (WFS) was associated with subsequent LTSA-MD, adjusting for all covariates (HR 1.60; 95% CI 1.10–2.16). Both high WTFC (1.64; 1.15–2.23), and high FTWC (1.43; 1.02–2.00) increased the probability of LTSA-MD in the full model. When participants with prior mental disorder were excluded, the association between poor WFS and WTFC with LTSA-MD retained while that between FTWC and LTSA-MD attenuated; however, two items of the FTWC were still associated with LTSA-MD: ‘*Family worries and problems distract you from your work’* and ‘*Family matters prevent you from sleeping enough to do your job well’.* Of the WTFC items, the following remained associated with LTSA-MD: ‘*Problems at work make you irritable at home’* and ‘*Your job takes so much energy you do not feel up to doing things that need attention at home’.* The experience of decreased time for work or family did not associate with LTSA-MD.

**Conclusions:**

. Among female municipal employees, dissatisfaction with combining work and family and both work-to-family and family-to-work conflicts were associated with subsequent long-term sickness absence due to mental disorders.

**Supplementary Information:**

The online version contains supplementary material available at 10.1186/s12889-023-16075-y.

## Introduction

Conflicts between the demands of work and family (WFC) may contribute to the occurrence of mood and anxiety disorders [1–5], as well as to sickness absence (SA) [6–10]. Negative spillover between work and private life among female employees was linearly associated with incident depressive symptoms [11]. On the other hand, symptoms of depression may in turn increase the likelihood of WFC [12, 13]. It has been pointed out that while WFC and decreased mental health are often linked, longitudinal data do not always show robust relationships [14, 15]. Moreover, many studies have used self-reported health outcomes.

Work can interfere with family responsibilities and vice versa [16, 17]. Conflict arises when the requirements of one role make it difficult to fulfill those of another [18, 19]. In their meta-analytic review of 178 samples, Michel et al. [20] found that various family role stressors (e.g., number of children/dependents, parental demands, role overload) preceded family-to-work conflicts, while work characteristics and stressors (e.g. task variety, job autonomy, work time demands, role conflict) preceded work-to-family conflicts, in addition to personality characteristics (internal locus of control, negative affectivity).

It has also been found that family-to-work conflicts are more common when children are under school age and work-to-family conflicts when they are in school age [21]. Caring for children and providing informal care increase the perceived work-life conflict according to analyses of the European Working Conditions Survey supplemented with macro-data on work-family facilities [22]. Shift workers have more difficulty combining work and family responsibilities than day workers [23]. A review of 11 intervention studies showed that work flexibility in terms of work time and other arrangements, and support from supervisors, decreased work-family conflict [24].

Mental disorders are nowadays the most common cause of medically certified SA in Finland [25] and in many other countries [26]. The occurrence of SA due to depression has increased annually especially among women [25, 27]. SA is often the first sign of reduced work ability and, if repeated and prolonged, easily leads to an early disability pension [28, 29]. There is a need to know whether WFC increases the risk for SA due to mental disorders. However, only a few studies have reported on the issue, and with contradictory results.

A 9-year follow-up study found an increased risk of SA due to non-psychotic psychiatric disorders among those who had simultaneously high levels of work and family demands due to several work-related stress factors and dependents [30]. However, a prospective twin-study with register-based diagnoses of SA [14] showed that among women, WFC was not associated with higher odds between SA and stress-related or other mental disorders after adjustment for self-rated health and familial factors (i.e., genetics and shared environment), while among men, there was an association between WFC and SA due to stress-related causes.

Based on recent systematic reviews and meta-analyses, exposure to psychosocial stressors at work increases the risk of SA due to a mental disorder [31, 32]. Further, consistent evidence exists that female gender, lower educational level, smoking, poor self-rated general health, higher symptom severity, co-morbidity, and previous absenteeism are associated with SA in people with a mental disorder [31]. An elevated risk of SA due to mental disorders has been reported among employees in welfare service occupations (within health care, education, and social services), where workers are mostly women [32].

Although work-family conflicts are recognized sources of mental distress, there is a shortage of studies that focus on their associations with SA due to mental disorders. Moreover, it is unclear whether the two directions of effect, i.e., work-to-family and family-to-work conflicts, differ in their association with mental disorders [15]. The present work adds to this literature by studying in detail both directions of a potential effect, in addition to overall satisfaction with combining work and family.

In this follow-up study, we investigated whether conflicts between work and family were associated with the occurrence of long-term SA due to mental disorders (LTSA-MD) among female municipal employees, working predominantly in welfare service occupations. We made use of several indicators of WFC: a single-item measure of overall satisfaction with combining work and family, which we called work-family satisfaction (WFS), a composite variable on the conflicts caused by work to the family (WTFC) and, conversely, the conflicts caused by family to work (FTWC). In addition, we investigated the components of the composite variables separately to get more detailed information about the factors that associate with conflicts. Several potentially confounding factors related to sociodemographic factors, family composition, working conditions, and overall self-rated health were adjusted for in the analyses. In a second set of analyses, we excluded employees who reported having earlier a mental disorder diagnosed by a physician.

## Materials and methods

### Study cohort

This study is part of the ongoing Helsinki Health Study (HHS) with phase 1 questionnaire surveys conducted in 2000 − 2002 with the response rate of 67% (men 60%, women 69%). The cohort initially included 8960 employees of the City of Helsinki, Finland, 7154 women and 1790 men, aged 40, 45, 50, 55, and 60 years. The aim of the HHS is to map and follow up the health and well-being of ageing municipal employees. Data collection and cohort characteristics have been presented in detail elsewhere [33].

Since the measures on conflict between work and the family were first included in 2001, only the data from surveys 2001 and 2002 were used in this study. Thus, our baseline data comprised 5819 participants of which 4674 were women (80%, response rate 69%). Based on preliminary analyses, we decided to exclude men from the study, since their number was not large enough for reliable results. Due to the general retirement age of 63 years in municipal employment during the follow-up, we excluded participants aged 60 from the study because of their short remaining work career. In addition, this age group was more likely than the others to have no family (11% vs. 6%). Thus, our data comprised 4118 women (response rate 69%), of these 3043 had given their written consent to the linkage of survey data to register data using their personal identity code.

Women in Finland usually work full time and part time work is rare. Thus, we made a restriction to those who worked for at least 30 h per week. As conflicts between work and family life are only relevant to those who are working and have a family, participants who reported not having a family were not considered. The final study populations included 2386 women. The description of the data is presented in detail using the flow-chart, which can be found in the Additional file 1.

### Outcome variable

The data for the outcome variable were received from the registers of the Social Insurance Institution, Finland, for the years 2004 − 2010. They consisted of the start and end dates of SA periods, if any, per year for each person, and included medically certified diagnostic labels for the periods. The outcome variable was defined as the time to the first SA spell that had lasted for at least 12 days due to a mental or behavioral disorder (ICD-10: F00 − F99). There were altogether 404 such cases.

### Other register data

In additional analyses, we used register data from the employer’s personnel register. The data comprised long-term sickness absence spells without diagnoses from 1 to 2000 to 31 Dec 2003. The participants had given their written consent also to the linkage of these data to the survey. Information of retirement from work was received from the Finnish Centre for Pensions and data on deaths from Statistics Finland from 1 to 2000 to 31 Dec 2010.

### Compensation scheme for SA in Finland

Employees aged 16 to 67 living in Finland and who are not retired are entitled to compensation for the loss of income during SA after the employer’s payment period has elapsed. The Social Insurance Institution pays the allowance, usually starting from the 12th calendar day of getting sick [25]. The employee can receive compensation up to 300 days per illness over a period of two years. If work incapacity continues longer, the employee can be granted either a temporary or a permanent disability pension.

### Indicators of work-family conflict

#### Work-family satisfaction (WFS)

Participants were asked: “*How satisfied are you with combining work and family*?” The response options were as follows: very satisfied, somewhat satisfied, neither satisfied nor dissatisfied, somewhat dissatisfied, and very dissatisfied. These were further classified in three categories: (1) satisfied, (2) neither satisfied nor dissatisfied, and (3) dissatisfied.

### Work-to-family conflicts (WTFC) and family-to-work conflicts (FTWC)

The indicators of WTFC and FTWC were originally presented by Grzywacz and Marks in a study of midlife development conducted in the US [16]. The item “Your work involves a lot of travel away from home” was omitted from the WTFC score, because such work was rare in the sample and according to a previous analysis [34] in the present study material, this item reduced the reliability of the WTFC variable.

### Work-to-family conflicts (WTFC)

WTFC were inquired as follows: “*How much do your work duties interfere with your family life*?“ The items were: (1) “*Your job reduces the amount of time you can spend together with your family*”, (2) “*Problems at work make you irritable at home*”, and (3) *“Your job takes so much energy you do not feel up to doing things that need attention at home*”. For all items the response options were 1 not at all, 2 to some extent, 3 a great deal and 4 I do not have a family.

### Family-to-work conflict (FTWC)

FTWC was inquired as follows: “*To what extent does your family life and responsibility for the family interfere with the performance of your work duties*?“. The items were: (1) “*Family matters reduce the amount of time you can devote to your job*”, (2) “*Family worries or problems distract you from your work*”, (3) “*Family matters prevent you from sleeping enough to do your job well*”, and (4) “*Family obligations reduce the time you need to relax or be by yourself*”. The response options were the same as above for the WFTC.

Before constructing the composite variables of WTFC and FTWC, correlations between the items were tested. The standardized Chronbach’s alpha coefficient (internal consistency) was 0.65 for the indicator of WTFC, and 0.73 for FTWC. When constructing these composite variables, we excluded those who chose the response option “I have no family”. The three items on WTFC were combined using the MAX function of the SAS software [35]. The function constructed a new variable based on the highest scores of the responses to these items and ranked the participants to classes like those of the original items (1 = not at all, 2 = to some extent, and 3 = a great deal). The FTWC was constructed similarly based on the four items.

### Covariates

We selected the covariates based on the literature that has suggested factors potentially confounding the association between work-family conflicts and sickness absence.

*Sociodemographic factors*. Age: 40, 45, 50 and 55 years; marital status: (1) married/cohabiting, (2) single (unmarried/divorced/widowed); number of children up to the age of 18 living in the same household: (1) 0, (2) 1, and 3) > 1; education: (1) high (bachelor’s, master’s and doctor’s degree), (2) basic to secondary (comprehensive-, upper secondary-, and vocational school).

*Self-rated health*. “*In general, would you say that your health is: excellent, very good, good, moderate or bad?*” The options were classified in two categories: 1) at least good and 2) < good.

*History of mental disorders*. The participants were asked whether a physician had ever diagnosed them to have any of the following (yes, no): depressive disorder, anxiety, or other mental disorder. The replies were combined into one variable called “*Prior mental disorder”* with two alternatives (no, yes). This variable was used in the analyses to limit participants to those who did not have a prior history of mental disorder.

*Work-related factors.* Questions about physical and mental strenuousness at work were as follows: “*How strenuous or light do you consider your work to be* a) *physically*, b) *mentally*?” The response options to both questions were very light, quite light, quite strenuous, and very strenuous, which were further classified to two categories: (1) light and (2) strenuous. Work-time schedule had initially the following response options: regular day work, regular night work, shift work without night work, shift work including night work, day work with night-time service, and other. Categories were merged as follows: (1) regular day job, (2) other.

### Statistical analyses

The association between work-family interference at baseline with the first occurrence of SA spell lasted at least 12 days due to a mental disorder was analysed using the proportional hazards model (Cox regression). The follow-up started on 1 Jan 2004 and ended on 31 Dec 2010. The proportional hazards assumption for all variables was tested by a Kolmogorov-type supremum test. The outcome variable was the number of days counted from the beginning of the follow-up to the first SA spell due to a mental disorder, retirement, death, or end of follow-up on 31 December 2010, whichever occurred first.

The associations with the first medically certified SA spell due to a mental disorder (LTSA-MD) were analysed for the overall variable of work-family satisfaction (WFS), two composite variables of work-to-family conflicts (WTFC) and family-to-work conflicts (FTWC), as well as their components separately. The categories “to some extent” and “a great deal” were combined in the multivariable models due to few observations.

We first calculated the unadjusted (crude) hazard ratios (HR) for all variables (Tables 1 and 2) among all participants. Thereafter two models were analysed. In model 1 the HRs were adjusted for age, marital status, number of children, education, work-time schedule, and perceived physical and mental strenuousness at work (separately). In model 2, self-rated health was additionally included as a covariate. These analyses were made for all participants and separately for those who did not have a prior history of mental disorder. All analyses were performed using SAS software package (version 9.4; SAS Institute, Inc, Cary, North Carolina).

**Table 1:** Background characteristics and medically certified sickness absence due to a mental disorder (LTSA-MD).

	All women N = 2386	LTSA-MD^1^ N = 404
	N (%)	N (%)
Background factors		
*Age*, years (mean)	47.5	46.8
40	564 (23.6)	105 (26.0)
45	651 (27.3)	114 (28.2)
50	580 (25.3)	116 (28.7)
55	591 (24.8)	69 (17.1)
*Marital status*		
Married/cohabiting	1706 (71.5)	261 (64.6)
Single	680 (28.5)	143 (35.4)
*Education*		
High	1429 (59.9)	220 (54.5)
Secondary/basic	957 (40.2)	184 (45.5)
*Number of children (≤ 18 yrs) in the same household*		
0	1189 (49.8)	210 (52.0)
1	557 (23.4)	102 25.2)
> 1	640 (26.8)	92 (22.8)
*Self-rated health*		
At least good	1938 (81.2)	283 (70.0)
< good	448 (18.8)	121 (30.0)
*Prior mental disorder*		
No	2040 (85.5)	281 (69.6)
Yes	346 (14.5)	123 (30.4)
*Work-time schedule*		
Regular day job	1892 (79.3)	300 (74.3)
Other	494 (20.7)	104 (25.7)
*Perceived physical strenuousness at work*		
Light	1515 (63.5)	245 (60.6)
Strenuous	871 (36.5)	159 (39.4)
*Perceived mental strenuousness at work*		
Light	579 (24.3)	79 (19.6)
Heavy	1807 (75.7)	325 (80.4)

^1^ ICD-10: F00 − F99, Helsinki Health Study

**Table 2:** Explanatory variables in association with sickness absence due to a mental disorder (LTSA-MD).

	All women N = 2328− 2386	LTSA-MD^1^ N = 392− 404	Unadjusted association^2^	
	N (%)	N (%)	HR	95% CI
Work-family satisfaction (WFS)				
*“How satisfied are you in combining work and family?”*				
Satisfied	1887 (81.1)	288 (73.5)	1	
Neither satisfied nor dissatisfied	231 (9.9)	47 (12.0)	1.42	1.04 − 1.93
Dissatisfied	210 (9.0)	57 (14.5)	1.88	1.41 − 2.50
Work-to-family conflicts score (WTFC)				
Not at all	443 (18.6)	57 (14.1)	1	
To some extent	1489 (62.4)	243 (60.2)	1.28	0.96 − 1.70
A great deal	454 (19.0)	104 (25.7)	1.88	1.36 − 2.60
Components:				
“*Your work reduces the time you can spend together with your family?“*				
Not at all	1060 (44.5)	172 (42.8)	1	
To some extent	1053 (44.2)	177 (44.0)	1.01	0.82 − 1.25
A great deal	269 (11.3)	53 (13.2)	1.19	0.88 − 1.63
“*Problems at work make you irritable at home?“*				
Not at all	937 (39.4)	127 (31.6)	1	
To some extent	1263 (53.1)	225 (56.0)	1.34	1.08 − 1.66
A great deal	180 (7.6)	50 (12.4)	2.18	1.57 − 3.03
“*Your job takes so much energy you do not feel up to doing things that need attention at home?“*				
Not at all	1039 (43.7)	140 (35.8)	1	
To some extent	1144 (48.1)	216 (53.7)	1.49	1.21 − 1.85
A great deal	194 (8.2)	46 (11.4)	2.03	1.46 − 2.83
Family-to-work conflicts score (FTWC)				
Not at all	1120 (46.9)	168 (41.6)	1	
To some extent	1027 (43.0)	183 (45.3)	1.17	0.95 − 1.44
A great deal	239 (10.0)	53 (13.1)	1.47	1.08 − 2.00
Components:				
“*Family matters reduce the amount of time you can devote to your job?“*				
Not at all	1940 (81.5)	328 (81.4)	1	
To some extent	398 (16.7)	65 (16.1)	0.92	0.71 − 1.20
A great deal	42 (1.8)	10 (2.5)	1.38	0.74 − 2.58
*“Family worries and problems distract you from your work?“*				
Not at all	1864 (78.2)	284 (70.7	1	
To some extent	494 (20.7)	108 (26.9)	1.47	1.18 − 1.83
A great deal	25 (1.1)	10 (2.4)	2.83	1.51 − 5.32
“*Family matters prevent you from sleeping enough to do your job well?”*				
Not at all	1907 (80.1)	289 (71.5)	1	
To some extent	435 (18.3)	103 (25.5)	1.66	1.32 − 2.07
A great deal	39 (1.6)	12 (3.0)	2.13	1.20 − 3.80
*“Family obligations reduce the time you need to relax or be by yourself?”*				
Not at all	1325 (55.7)	215 (53.6)	1	
To some extent	857 (36.0)	141 (35.2)	0.98	0.79 − 1.21
A great deal	198 (8.3)	45 (11.2)	1.40	1.02 − 1.94

^1^ ICD-10: F00 − F99, ^2^ Cox proportional hazards regression, hazard ratios (HR) with their 95% confidence intervals (CI), Helsinki Health Study

Sensitivity analyses.

### Comparison of the age distribution

We studied the representativeness of our sample by age (Additional file 2, Table 1x). We compared the age-group-specific distribution of the data in this study with the distribution of the original data (without those aged 60 years) and found that the 40- and the 45-year-old participants were slightly overrepresented (by 1% and 3%, respectively), while the 55-year-old were underrepresented by 4%. The reason for the deviations was probably the exclusion of those without a family because the questions were intended for people with families. While the share of persons without a family in the total data was 6%, it was 5% for the 40-year-old, 4% for the 45-year-old, 7% for the 50-year-old and 9% for the 55-year-old participants.

### Long-term sickness absence (LTSA) due to any diagnosis

We constructed a variable to describe whether the participant had had at least one sickness absence of more than 11 days between 1 and 2000 and 31 Dec 2003. The information from the employer’s personnel register data was available for 2351 participants. These data comprise all sickness absence spells before the start of our follow-up without diagnostic information. There were altogether 659 such sickness absence spells. The cross-tabulation revealed that such preceding LTSA were only linked with FTWC (Additional file 2, Table 2x). We also conducted further analyses on the association between FTWC and its components with LTSA-MD in which the variable on any previous LTSA (no/yes) was added to the list of covariates, but this had only a negligible impact on the results.

### Additional models

Moreover, we present four models that examine more closely the effect of the covariates on the associations of WFS, WTFC and FTWC with LTSA-MD (Additional file 2, Table 3x). In model A, only age is included as a covariate, in model B, age and education, in model C, the number of children in the same household in addition to the variables above, and in model D, age, education, work-time schedule, and mental and physical strenuousness at work were included.

**Table 3:** Work-family satisfaction in association with sickness absence due to a mental disorder (LTSA-MD^1^).

	All women, N = 2328 cases, N = 392					Those who had no prior mental disorder, N = 1990, cases, N = 271						
Adjusted for:	Model 1: age, marital status, number of children, education, work-time schedule, mental and physical strenuousness at work		Model 2: Model 1 + self-rated health		Model 1: age, marital status, number of children, education, work-time schedule, mental and physical strenuousness at work			Model 2: Model 1 + self-rated health				
	HR^2^	95% CI	HR^2^	95% CI		HR^2^	95% CI		HR^2^	95% CI		
Work-family satisfaction (WFS)												
*“How satisfied are you in combining work and family?”*												
Satisfied	1		1			1			1			
Neither satisfied nor dissatisfied	1.31	0.96 − 1.80	1.16	0.85 − 1.59		1.31	0.89 − 1.94		1.22	0.83 − 1.81		
Dissatisfied	1.88	1.40 − 2.52	1.60	1.19 − 2.16		1.77	1.22 − 2.57		1.60	1.10 − 2.34		

^1^ ICD-10: F00 − F99, ^2^ Cox proportional hazards regression, hazard ratios (HR) with their 95% confidence intervals (CI), Helsinki Health Study

## Results

Table 1 presents the background characteristics of the participants. The mean age of the participants was 48 years. They were usually married or cohabiting (72%), while 23% had one child and 27% more than one child living in the same household. All participants worked at least 30 h per week and 79% had a regular day job. About one third reported that their work was physically heavy and three out of four experienced it to be mentally heavy (quite heavy: 63%, very heavy: 13%).

During the 7-year follow-up, 17% of the participants had at least one SA period due to a mental disorder. Of these 65% were granted due to an affective disorder (F30 − F39) and 30% due to a neurotic, stress-related or somatoform disorders (F40 − F48). Among those (15%) who had been diagnosed by a physician to have a mental disorder at some point before the baseline survey, 30% had a LTSA-MD during the follow-up (Table 1).

Table 2 presents the distribution of explanatory factors and their unadjusted associations with LTSA-MD. Of all respondents, 9% were dissatisfied with combining work and family (WFS) when inquired with one overall question. Dissatisfaction was more common in younger age groups than in older ones: 13% in 40-year-old, 11% in 45-year-old, 7% in 50-year-old and 5% in 55-year-old (data not shown).

According to the composite variables, 19% reported high WTFC and 10% high FTWC. Those who reported that they experienced high WTFC had LTSA-MD more often (26%) than average (19%). The corresponding figures were 13% and 9% among those women who reported high FTWC.

Being dissatisfied with combining work and family, or having high conflicts as based on the WTFC, increased equally the risk of LTSA-MD during follow-up. These relationships were stronger than that between FTWC and LTSA-MD.

While most items of the WTFC and FTWC were associated with subsequent LTSA-MD, the experience of reduced time with family because of work, and conversely, of reduced time for work because of family responsibilities, were not. This was true although 56% of the participants felt that work reduced the time they could spend together with family, and 19% that family matters reduced the time they could devote to work.

### Work-family satisfaction (WFS) and subsequent LTSA-MD in multivariable models

Table 3 presents associations between WFS with LTSA-MD among all women as well as among those who did not report any prior mental disorder. Two models were calculated: the first model was adjusted for age, marital status, living with children, education, work-time schedule, and physical and mental strenuousness at work. The second model was further adjusted for self-rated health.

Among all women, poor WFS was associated with SA both in the first model (HR 1.88; 95% CI 1.40–2.52) and adjusting also for self-rated health (1.60; 1.19–2.16). When the participants with a prior mental disorder were removed from the analyses, the associations retained: the HR in the first model was 1.77 (1.22–2.57) and in the second 1.60 (1.10–2.34).

### Work-to-family conflicts (WTFC) and subsequent LTSA-MD in multivariable models

Table 4 presents the two models of the WTFC score and its three components in association with LTSA-MD, using the same modeling sequence as above. Among all women (model 1), compared to those with no conflicts, high WTFC was associated with subsequent LTSA-MD with the HR of 2.04 (1.44–2.87), while allowing further for self-rated health (model 2), the HR was 1.64 (1.15 − 2.23).

**Table 4:** Work-to-family conflicts in association with sickness absence due to a mental disorder (LTSA-MD^1^).

	All women, N = 2377 − 2386, cases, N = 401 − 404					Those who had no prior mental disorder, N = 2032 − 2040, cases, N = 279 − 281				
Adjusted for:	Model 1: age, marital status, number of children, education, work-time schedule, mental and physical strenuousness at work		Model 2: Model 1 + self-rated health		Model 1: age, marital status, number of children, education, work-time schedule, mental and physical strenuousness at work			Model 2: Model 1 + self-rated health		
	HR^2^	95% CI	HR^2^	95% CI	HR^2^		95% CI	HR^2^	95% CI	
Work-to-family conflicts score (WTFC)										
Not at all	1		1		1			1		
To some extent	1.37	1.02 − 1.85	1.25	0.93 − 1.69	1.11		0.79 − 1.56	1.04	0.74 − 1.47	
A great deal	2.04	1.44 − 2.87	1.64	1.15 − 2.23	1.81		1.22 − 2.68	1.58	1.05 − 2.37	
Components:										
“*Your job reduces the amount of time you can spend together with your family?”*										
Not at all	1		1		1			1		
To some extent/a great deal	1.12	0.91 − 1.39	1.07	0.86 − 1.32	1.07		0.83 − 1.39	1.03	0.78 − 1.34	
“*Problems at work make you irritable at home?”*										
Not at all	1		1		1			1		
To some extent/a great deal	1.52	1.22 − 1.90	1.38	1.10 − 1.73	1.40		1.08 − 1.82	1.32	1.01 − 1.71	
“*Your job takes so much energy you do not feel up to doing things that need attention at home?”*										
Not at all	1		1		1			1		
To some extent/a great deal	1.55	1.25 − 1.92	1.35	1.09 − 1.68	1.35		1.05 − 1.73	1.24	0.96 − 1.60	

^1^ ICD-10: F00 − F99, ^2^ Cox proportional hazards regression, hazard ratios (HR) with their 95% confidence intervals (CI), Helsinki Health Study

When participants who had a prior mental disorder, were left out of the analyses, the HR of high conflicts in model 1 was 1.81 (1.22–2.68). Further adjustment for self-rated health in model 2 weakened the associations somewhat, but among those with high WTFC the association persisted (1.58; 1.05–2.37).

WTFC components. When the individual items of the WTFC score were studied separately among all women, it appeared that agreement with the items of *‘Problems at work make you irritable at home’* (1.38; 1.10 − 1.73) and *‘Your job takes so much energy you do not feel up to doing things that need attention at home’* (1.35; 1.09 − 1.68) were still associated with SA after all adjustments (Table 4). However, the latter association attenuated when women with earlier mental disorder were left out of the analyses.

### Family-to-work conflicts and subsequent LTSA-MD in multivariable models

Table 5 presents accordingly two models of the composite variable of FTWC, and its four components, in association with LTSA-MD. Results are shown first among all women and secondly among those who did not have a prior mental disorder.

**Table 5:** Family-to-work conflicts in association with sickness absence due to a mental disorder (LTSA-MD^1^).

	All women, N = 2377 − 2386, cases = 401 − 404					Those who had no prior mental disorder, N = 2032 − 2040, cases = 279 − 281				
Adjusted for:	Model 1: age, marital status, number of children, education, work-time schedule, mental and physical load at work		Model 2: Model 1 + self-rated health		Model 1: age, marital status, number of children, education, work-time schedule, mental and physical load at work			Model 2: Model 1 + self-rated health		
	HR^2^	95% CI	HR^2^	95% CI	HR^2^		95% CI	HR^2^	95% CI	
Family-to-work conflicts score (FTWC)										
Not at all	1		1		1			1		
To some extent	1.34	1.07 − 1.67	1.20	0.96 − 1.50	1.12		0.86 − 1.46	1.04	0.80 − 1.36	
A great deal	1.72	1.24 − 2.39	1.43	1.02 − 2.00	1.24		0.80 − 1.94	1.11	0.71 − 1.74	
Components:										
“*Family matters reduce the amount of time you can devote to your job?”*										
Not at all	1		1		1			1		
To some extent/a great deal	1.03	0.79 − 1.33	0.95	0.73 − 1.24	0.75		0.53 − 1.07	0.73	0.51 − 1.04	
*“Family worries or problems distract you from your work?“*										
Not at all	1		1		1			1		
To some extent/a great deal	1.53	1.23 − 1.91	1.38	1.11 − 1.72	1.40		1.06 − 1.84	1.33	1.00 − 1.75	
*“Family matters prevent you from sleeping enough to do your job well?”*										
Not at all	1		1		1			1		
To some extent/a great deal	1.72	1.38 − 2.14	1.54	1.23 − 1.93	1.45		1.09 − 1.93	1.38	1.03 − 1.85	
*“Family obligations reduce the time you need to relax or be by yourself?”*										
Not at all	1		1		1			1		
To some extent/a great deal	1.18	0.95 − 1.47	1.06	0.85 − 1.32	1.01		0.78 − 1.32	0.94	0.72 − 1.23	

^1^ ICD-10: F00 − F99, ^2^ Cox proportional hazards regression, hazard ratios (HR) with their 95% confidence intervals (CI), Helsinki Health Study

The fully adjusted (model 2) HR of LTSA-MD among those who had high FTWC was 1.43 (1.02 − 2.00) compared to women with no conflicts. When similar analyses were performed only among those without a prior mental disorder, FTWC in general was no more associated with LTSA-MD.

FTWC components. In the fully adjusted model, for those 22% who experienced that *“family worries or problems distract you from your work "* to some extent or a great deal, the HR of SA was 1.38 (1.11 − 1.72) compared to the group with no such worries. Similarly, those 20% who agreed to the assertion “*family matters prevent you from sleeping enough to do your job well”*, had an increased risk of LTSA-MD (1.54; 1.23–1.93). After restricting the analyses to those without a prior mental disorder, the respective HRs were 1.33 (1.00 − 1.75) and 1.38 (1.03–1.85). The items *“Family matters reduce the amount of time you can devote to your job?“* or *“Family obligations reduce the time you need to relax or be by yourself?”* did not associate with LTSA-MD.

The four supplementary models for WFS, WTFC and FTWC (Additional file 2, Table 3x) showed that adjustment for education or marital status and number of children, strengthened the associations, whereas adjustment for work-time schedule and physical and mental strenuousness at work, attenuated the associations.

## Discussion

We studied associations of conflicts between the demands of work and family with subsequent long-term sickness absence due to mental disorders (LTSA-MD) among women in municipal occupations. The study design was prospective and medically certified, register-based information on absences were used as the outcome measure. High WTFC were perceived by every fifth participant, redoubling the risk of LTSA-MD when age, marital status, number of children, level of education, perceived mental and physical strenuousness at work, and work-time schedule were controlled for. Our results also showed that a single question on overall satisfaction in combining work and family was linked with LTSA-MD in a similar manner as WTFC. On the other hand, high FTWC were less common and associations with LTSA-MD weaker, in line with earlier studies that considered overall SA [3, 6, 8, 36].

We further examined the effect of conflicts as based on the single items of the composite scores of WTFC and FTWC. Of the items in the WTFC, increased irritability and energy depletion, and of the items of the FTWC, concentration difficulties at work due to family worries and disturbed sleep, were associated with LTSA-MD. Contrary to this, it seemed that time constraints were not at the core of the relationships, as neither job reducing the time for family nor family matters reducing the time for work or for relaxation, were associated with the outcome. Our findings thus underline the role of psychological reactions and mental strain [37], as well as the importance of sleep loss, as components of work-family conflicts. In line with this, a recent study among employed parents in the IT industry found that affective reactivity to WTFC, assessed over 8 consecutive days, was associated with poorer sleep quality and distress [38]. Further, poorer sleep quality was associated with lower productivity and higher WTFC the next day [39].

To further clarify the studied relationship, we were able to limit a second set of analyses to participants without a previous diagnosis of mental disorder by a physician, based on self-report. After this exclusion the associations of FTWC attenuated considerably, while those of WTFC persisted. However, the possibility of recall bias must also be considered. Yet our analyses suggest that FTWC was more strongly related to participants prior mental health.

### Interpretation of results

Earlier studies with which to compare our results are few. A large cohort study [40] investigated the direct and indirect effects of psychosocial working conditions on mental health related SA lasting for more than 42 days. Work-family conflict was assessed as job demand-family interference by a 7-item sum score. High interference was associated with subsequent SA due to mental disorders, adjusted for sociodemographic and work-related factors, and previous long-term SA due to mental disorders. It was also found that distress mediated the effect of job demand-family interference on SA. The results are in line with our findings, even though the occupational sectors were different (included were e.g., industry, commercial business, agriculture, and public services) from ours where all participants were employed in public services.

The Swedish twin study [14] found that the odds ratios of work-family conflict with SA due to stress-related and other mental disorders in women (but not in men) were near unity after adjustment for self-rated health. In our material, however, the association of WTFC persisted even with adjustment for self-rated health. This was also true for the FTWC score in the total material. Taking overall self-rated health into the model could even be argued as leading to overadjustment in the present context of mental disorders. The difference between our results and those of Svedberg et al. [14] may be due to differences of measuring work-family conflicts as well as participants’ characteristics. In our study the participants were already middle-aged at baseline, which could have bearing on the pattern of sources of conflict, and they worked in welfare professions, where employees may be at a particular risk for mental disorders and related SA [27].

Among a middle-aged cohort of workers, the number of work stresses and of dependents in the family were studied in relation to SA due to mental disorders over 9 years [30]. High co-occurring work and family demands were strongly associated with subsequent SA due to mental disorders particularly in women. Among other covariates, this study controlled for depressive symptoms at baseline.

In our study three out of four participants experienced their work to be mentally strenuous. However, only 17% of them had LTSA-MD during the 7-year follow-up. Previous studies have shown that psychosocial stress factors at work are linked with mental disorders and the sick leave due to them [41]. It seems that conflicts that work life causes to the family sphere, while reflecting the psychosocial load factors of work, are nevertheless partly independent of these.

### Methodological considerations

We used a large representative sample of Finnish female public sector employees in full-time work. We were able to utilize official comprehensive and valid national register-based data on medically certified LTSA-MD, retirement, and death, eliminating response and recall biases. The seven-year follow-up period was long enough to provide a reliable number of cases with LTSA-MD.

The original Helsinki Health Study phase 1 survey had a good response rate among women (69%), and of our sample, 74% had given their written consent to the linkage of survey data to register data. As the public sector employees are mostly women and the number of men in the survey data was limited, we restricted the analyses to women.

The measures to assess WFC were derived and adopted from the US National Study of Midlife Development [16]. They have also been used e.g., in the British Whitehall II Study [42] and in the Japanese Civil Servants Study [43]. Many HHS-studies have shown these indicators to be suitable for the study of work-family conflicts (e.g., [44], [45], [46]).

The Cronbach’s alpha of the WTFC indicator was 0.65 and that of the FTWC, 0.73. The latter figure may be considered sufficient, but the former could be better. A modest alpha means that the subjects’ answers to the questions have not been completely consistent, which can reduce the reliability of the indicator and weaken the associations. However, the WTFC showed quite consistent associations with LTSA-MD.

We considered a variety of possible confounders. Work that is perceived as mentally or physically strenuous can be assumed to be linked to both WFC and LTSA-MD. Yet the inclusion of work factors in the models may lead to overadjustment. However, adjustment of perceived mental and physical strenuousness at work had some, but not, remarkably effect on the results (Additional file 2, Table 3x). In a recent study, these same work-related indicators, that we used, showed quite strong associations with subsequent antidepressant medication use in young adults [47]. The authors of that study explored, that the indicator of perceived mental strenuousness of work showed a strong correlation with the Karasek’s job demands measure. In turn, the indicator measuring the perceived physical strenuousness of work was most strongly correlated with the long daily duration of heavy work [47].

The study has also some other limitations to be considered. The follow-up started on the 1st of Jan 2004 because LTSA data with diagnoses were not available for research use before that date. In additive sensitivity analyses, we included any LTSA for four years before the start of the follow-up in the models. We found that previous LTSA were only linked to the FTWC and including them in the models had a minor impact on the results (data not shown). Anyway, this lack of data may have affected the results.

The national registers do not provide information on short-term absences with diagnoses, and we could not assess the association of work-family conflicts with SA less than 12 workdays. We can assume that the outcome mostly represents more serious mental health problems as a physician had set the diagnosis and ordered a long-term sickness absence. However, possibly not all participants have been able or willing to have a long absence from work despite a mental health problem. Moreover, all participants were in full-time work at the beginning of the follow-up. Thus, the healthy worker effect may have attenuated the associations between WFC and LTSA-MD.

Although the number of participants who gave their permission to the linkage between survey and register data was quite high, not all gave their consent. Non-response analysis showed only small differences in returning the questionnaire and consenting to register linkages by age, occupational class, income, type of employment contract and employment sector [48]. We also compared the age distribution of our data with the age distribution of the original data and observed only small differences in the proportions. Although municipal occupations involve many different tasks, the results may not be directly generalizable to other sectors of activity.

## Conclusions

Our results underline the importance of work-family conflicts as antecedents of SA due to mental disorders. We add to earlier studies more detailed information on the sources of conflicts reflecting both directions of association. Regarding work-to-family conflicts we found that psychological effects (irritation due to problems at work, energy loss) were associated with subsequent LTSA-MD, while reduced time available for family was not. On family-to-work conflicts we found that family worries causing distraction at work and not getting enough sleep to do the work well, were associated with subsequent LTSA-MD, while reduced time for relaxation was not. As demands of working life continue to increase and the population is ageing, reconciling work and family remain important issues, both in terms of working conditions and family policy.

## Electronic supplementary material

Below is the link to the electronic supplementary material.

Supplementary Material 1

Supplementary Material 2

## Data Availability

The dataset of the current study is not publicly available due to strict data protection laws and regulations. The Helsinki Health Study data are available exclusively to the members of the researcher group and can only be used for scientific purposes. Requests and collaboration initiatives can be directed to the Helsinki Health study group. Research group | Helsinki Health Study | University of Helsinki.

## Abbreviations

CI: Confidence interval
HR: Hazard ratio
ICD-10: International classification of diseases
LTSA: Long-term sickness absence due to any diagnosis
LTSA-MD: Long-term sickness absence due to mental disorders
SA: Sickness absence
WFS: Work-family satisfaction
WFC: Work-family conflict
WTFC: Work-to-family conflict
FTWC: Family-to-work conflict
