# Supplementary material for: Work-family conflicts and long-term medically certified sickness absence due to mental disorders – a follow-up study of female municipal employees
Source: BMC Public Health. 2023 Jun 13;23:1137. doi: 10.1186/s12889-023-16075-y (PMC10265818; doi:10.1186/s12889-023-16075-y)
Supplement: Supplementary file 1 — Supplementary Material 1 [file 12889_2023_16075_MOESM1_ESM.docx]

Additional file 1.

Available (N=2839)

Excluded: Part-time workers (N=171), working unknown (N=107)

Register linkage with sickness absence spells (N=3043)

Excluded: Deceased/disability pensioned (N=204)

Excluded: ≥ 60-year-old (N=556)

Helsinki Health Study (HHS)

Survey data from years 2001-2002 (N=5819): men and women aged 40-60 years

Excluded: Men (N=1145)

Women (N=4674)

Women 40-55-year-old (N=4118)

Excluded: No consent to register linkage on sickness absence spells (N=1075)

Have a family (N=2403)

Excluded: No family (N=158)

Final data (N=2368)

Full-time workers (N=2561)

Excluded: Missing data (N=35)

Figure 1. Flowchart of the study participants.
