# Supplementary material for: Work-family conflicts and long-term medically certified sickness absence due to mental disorders – a follow-up study of female municipal employees
Source: BMC Public Health. 2023 Jun 13;23:1137. doi: 10.1186/s12889-023-16075-y (PMC10265818; doi:10.1186/s12889-023-16075-y)
Supplement: Supplementary file 2 — Supplementary Material 2 [file 12889_2023_16075_MOESM2_ESM.docx]

Additional file 2/2.

Table 1x. Age distribution in the original data and in the data of this study.

| Age | Women who responded to the baseline survey (N=4118) | Women who gave their consent to the register linkage (N=3043) | Women eligible in this study (N=2386) |
| --- | --- | --- | --- |
|  | % | % | % |
| 40 | 23 | 23 | 24 |
| 45 | 24 | 25 | 27 |
| 50 | 24 | 24 | 24 |
| 55 | 29 | 29 | 25 |
| All | 100 | 100 | 100 |

Helsinki Health Study.

Table 2x. Long-term sickness absence (LTSA) before the follow-up by the outcome and explanatory factors.

|  |  | LTSA for any reasons in 2000−2003 (N=659) | |  |
| --- | --- | --- | --- | --- |
| N=2351 | All | No | Yes |  |
|  | % | (%) | (%) | p-value^1^ |
| Long-term sickness absence due to mental disorders (LTSA-MD) in 2004−2010 |  |  |  | <0.001 |
| No | 83.2 | 87.7 | 71.8 |  |
| Yes | 16.8 | 12.4 | 28.2 |  |
| Work-family satisfaction (WFS) |  |  |  | 0.184 |
| Satisfied | 81.0 | 81.6 | 79.3 |  |
| Neither satisfied nor dissatisfied | 9.9 | 9.2 | 11.7 |  |
| Dissatisfied | 9.1 | 9.2 | 8.9 |  |
| Work-to-family conflicts score (WTFC) |  |  |  | 0.788 |
| Not at all | 18.6 | 18.9 | 17.8 |  |
| To some extent | 62.5 | 62.3 | 62.8 |  |
| A great deal | 18.8 | 18.7 | 19.4 |  |
| Family-to-work conflicts score (FTWC) |  |  |  | 0.010 |
| Not at all | 47.0 | 48.6 | 42.8 |  |
| To some extent | 43.1 | 42.0 | 45.8 |  |
| A great deal | 10.0 | 9.4 | 11.4 |  |

^1^Pearson's chi-squared test. Helsinki Health Study.

Table 3x. WFS, WTFC and FTWC in association with LTSA-MD^1^ in four supplementary models.

| Adjusted for: | Model A: age | |  | Model B: age and education | |  | Model C: age, education, marital status, number of children | |  | Model D: age, education, work-time schedule, mental and physical load at work | |
| --- | --- | --- | --- | --- | --- | --- | --- | --- | --- | --- | --- |
|  | HR^2^ | 95 % CI |  | HR^2^ | 95 % CI |  | HR^2^ | 95 % CI |  | HR^2^ | 95 % CI |
| Work-family-satisfaction (WFS) |  |  |  |  |  |  |  |  |  |  |  |
| *How satisfied are you in combining work and family?* |  |  |  |  |  |  |  |  |  |  |  |
| Satisfied | 1 |  |  | 1 |  |  | 1 |  |  | 1 |  |
| Neither satisfied nor dissatisfied | 1.42 | 1.04−1.93 |  | 1.46 | 1.07−1.99 |  | 1.37 | 1.00−1.88 |  | 1.30 | 0.95−1.78 |
| Dissatisfied | 1.88 | 1.41−2.50 |  | 2.00 | 1.50−2.66 |  | 2.02 | 1.52−2.70 |  | 1.88 | 1.40−2.53 |
| Work-to-family conflicts score (WTFC) |  |  |  |  |  |  |  |  |  |  |  |
| Not at all | 1 |  |  | 1 |  |  | 1 |  |  | 1 |  |
| To some extent | 1.28 | 0.96−1.71 |  | 1.35 | 1.01−1.80 |  | 1.46 | 1.09−1.96 |  | 1.36 | 1.01−1.83 |
| A great deal | 1.89 | 1.37−2.62 |  | 2.06 | 1.48−2.86 |  | 2.30 | 1.65−3.20 |  | 2.03 | 1.44−2.86 |
| Family-to-work score (FTWC) |  |  |  |  |  |  |  |  |  |  |  |
| Not at all | 1 |  |  | 1 |  |  | 1 |  |  | 1 |  |
| To some extent | 1.16 | 0.94−1.44 |  | 1.21 | 0.98−1.50 |  | 1.34 | 1.07−1.67 |  | 1.29 | 1.04−2.34 |
| A great deal | 1.46 | 1.07−2.00 |  | 1.54 | 1.12−2.10 |  | 1.79 | 1.29−2.48 |  | 1.68 | 1.21−2.34 |

^1^ Long-term sickness absence due to mental disorder, ICD-10: F00−F99, ^2^ Cox proportional hazards regression, hazard ratios (HR) with their 95 % confidence intervals (CI). Helsinki Health Study.
